# Metabolic reprogramming in macrophage responses

**DOI:** 10.1186/s40364-020-00251-y

**Published:** 2021-01-06

**Authors:** Yang Liu, Ruyi Xu, Huiyao Gu, Enfan Zhang, Jianwei Qu, Wen Cao, Xi Huang, Haimeng Yan, Jingsong He, Zhen Cai

**Affiliations:** 1 Bone Marrow Transplantation Center, The First Afliated Hospital, School of Medicine, Zhejiang University, Hangzhou, Zhejiang China; 2 Institute of Hematology, Zhejiang University, Hangzhou, Zhejiang China; 3 Zhejiang Laboratory for Systems & Precison Medicine, Zhejiang University Medical Center, Hangzhou, Zhejiang China

**Keywords:** Macrophages, Metabolism, Glycolysis, Fatty acid synthesis, Fatty acid oxidation

## Abstract

Macrophages are critical mediators of tissue homeostasis, with the function of tissue development and repair, but also in defense against pathogens. Tumor-associated macrophages (TAMs) are considered as the main component in the tumor microenvironment and play an important role in tumor initiation, growth, invasion, and metastasis. Recently, metabolic studies have revealeded specific metabolic pathways in macrophages are tightly associated with their phenotype and function. Generally, pro-inflammatory macrophages (M1) rely mainly on glycolysis and exhibit impairment of the tricarboxylic acid (TCA) cycle and mitochondrial oxidative phosphorylation (OXPHOS), whereas anti-inflammatory macrophages (M2) are more dependent on mitochondrial OXPHOS. However, accumulating evidence suggests that macrophage metabolism is not as simple as previously thought. This review discusses recent advances in immunometabolism and describes how metabolism determines macrophage phenotype and function. In addition, we describe the metabolic characteristics of TAMs as well as their therapeutic implications. Finally, we discuss recent obstacles facing this area as well as promising directions for future study.

## Introduction

Macrophages are distributed in tissues throughout the body, and tissue-resident macrophages are derived from embryonic development and circulating monocytes. Despite their great diversity, macrophages play a major role in pathogen clearance via phagocytosis and contribute to proinflammatory processes, anti-inflammatory processes and tissue repair [1]. Macrophages are traditionally classified into two main groups: classically activated (M1) macrophages and alternatively activated (M2) macrophages [2]. M1 macrophages activated by lipopolysaccharide (LPS) with or without interferon-γ (IFN-γ) are proinflammatory and express inducible nitric oxide synthase (iNOS). In contrast, M2 macrophages activated by IL-4/IL-10 are more heavily involved in inflammation resolution, wound healing, helminth parasite resistance and expression of arginase 1 (Arg1) [3, 4]. Recent studies have revealed that macrophages possess distinct metabolic characteristics that correlate with their functional state, which is known as metabolic reprogramming. Here, we provide our perspective on recent developments related to the metabolic characteristics of macrophages, including arginine metabolism, glycolysis, the pentose phosphate pathway (PPP), the tricarboxylic acid (TCA) cycle, fatty acid metabolism, and glutamine metabolism and discuss their possible roles in immunity. Moreover, we describe the metabolic characteristics of tumor-associated macrophages (TAMs) as well as therapeutic implications. Finally, we discuss both recent obstacles and future research directions in metabolic reprogramming of macrophages.

Importantly, special attention is directed toward the metabolic disparity among macrophages of diverse origins, activated by diverse stimuli, and in diverse species [5–8]. Whether these macrophages exhibit associated differences remains to be investigated. In this review, we specify the sources, activators, and markers of the macrophages used in different studies.

## Metabolic regulation in different macrophage phenotypes

Generally, M1 macrophages express iNOS to produce nitric oxide (NO) from arginine, and display enhanced glycolytic metabolism, PPP, Fatty acid synthesis (FAS) and impaired TCA cycle and mitochondrial oxidative phosphorylation (OXPHOS). In contrast, M2 macrophages metabolize arginine by Arg-1, and are characterized by enhanced OXPHOS, FAS, and glutamine metabolism, and decreased PPP (Table 1). In this section, we will discuss the different intracellular metabolic pathways that regulate the polarization and function of macrophages (Table 2).

**Table 1:** Metabolic pathways in macrophages

	M1/M[LPS(±IFNγ)]	M2/M[IL-4/IL-10]	Contradictory observations
**Arginine metabolism**	**Metabolized arginine to NO and citrulline by iNOS**^**(9)**^	**Hydrolyzed arginine to ornithine and urea by arginase**^**(9)**^	
**Glycolysis**	**Glycolysis is enhanced**^**(21)**^	**Glycolysis is essential for M2 activation**^**(11,34,36,37)**^	**Glycolysis does not affect M2 activation**^**(38)**^
**PPP**	**Increased PPP pathway, providing NADPH for the production of ROS and NO**^**(25,41)**^	**Upregulation of CARKL, which is contributed to the suppression of PPP**^**(41)**^	
**OXPHOS**	**Reduced OXPHOS**^**(21)**^ **with the conditions of high mitochondrial membrane potential, M1 leads to reversal of the normal direction of electron flow causing RET at complex I, driving ROS production**^**(56)**^	**High OXPHOS**^**(21,34)**^	
**TCA cycle**	**Broken in two places — after citrate and after succinate**^**(40)**^	**An intact TCA cycle**^**(40)**^	
**FAS**	**FAS is increased and is contributed to the pro-inflammatory responses**^**(60-62)**^	**FAS fuels of FAO in M2 activation**^**(37)**^	
**FAO**		**Enhanced FAO is contributed to the activation of M2**^**(65,66)**^	**FAO is not essential during the M2 activation**^**(67,69)**^
**Glutamine metabolism**	**Glutamine can replesh succinate through “GABA shunt” and anaplerosis from a-ketoglutarate, and promotes the inflammatory response of macrophages**^**(53)**^	**Glutamine is contributed to the M2 activation**^**(40,71)**^	**Transiently deprived macrophage of glutamine has no effect for M1 polarization**^**(40)**^

**Table 2:** Roles of different metabolic profiles in macrophage phenotype and function. BMDMs, bone marrow-derived macrophages; MoDMs, human monocyte-derived macrophages; PMs, peritoneal macrophages

Experimental model(s)	Stimulation(s)	Regulator(s)	Effect(s)	Inhibitor(s)
***Arginine metabolism***				
**BMDMs/MoDMs**	**LPS/IFNγ**	**iNOS**	**NO blunts mitochondrial respiration and prevent M1 repolarization to the M2 phenotype**^**(11)**^	
**BMDMs**	**LPS/IFNγ**	**iNOS**	**NO contributes to ETC impairment and promote protective processes to mitigate NO-induced damage**^**(12)**^	
***Glycolysis***				
**RAW264.7**	**GLUT1 overexpression**	**GLUT1**	**GLUT1-OE macrophages enhance inflammatory cytokine release**^**(22)**^	**2DG(Glycolysis inhibitor)**
**PMs**	**Elicited by Brewer thioglycollate broth injection**	**glycolysis**	**Elicited macrophages have higher levels of glycolysis, which may be related to their increased phagocytic capacity**^**(23)**^	**2DG(Glycolysis inhibitor)**
**PMs /mouse** ** J774A.1 macrophages** **/BMDMs/THP1**	**LPS and ATP**	**HK1**	**HK1-dependent glycolysis is critical for NLRP3 inflammasome activation**^**(27)**^	**2DG(Glycolysis inhibitor)**
**BMDMs**	**LPS and ATP**	**HK**	**HK dissociation is sufficient to induce NLRP3 inflammasome activation**^**(28)**^	
**PMs /** **BMDMs**	**IFN or VSV**	**PFKFB3**	**PFKFB3-driven glycolysis selectively promotes the extrinsic antiviral capacity of macrophages**^**(29)**^	**PFK15(PFKFB3 inhibitor)**
**BMDMs/RAW264.7**	**tuberculosis (TB) infection**	**PFK-M**	**PFK-M dependent glycolysis drives host defense**^**(30)**^	
**BMDMs**	**LPS**	**GAPDH**	**GAPDH regulates TNFα production**^**(31)**^	
**BMDMs**	**LPS**	**PKM2**	**Activation of PKM2 promotes M1 polarization to the M2 macrophage and inhibites LPS-induced IL-1β**^**(32)**^	**DASA-58 and TEPP-46(PKM2 activators)**
**BMDMs/THP1/PMs**	**LPS+ATP or** **dA:dT**	**PKM2**	**PKM2-dependent glycolysis promotes NLRP3 and AIM2 inflammasome activation**^**(33)**^	**Shikonin (PKM2 inhibitor)**
***TCA cycle***				
**BMDMs/PMs/RAW264.7**	**LPS/IL-4**	**PDK1**	**Knockdown of PDK1 diminishes M1, whereas it enhances M2 activation**^**(34)**^	**2DG(Glycolysis inhibitor)**
**TEMPs/RAW264.7/in vivo**	**mild hypoxia /LPS**	**PDK1**	**PDK1 significantly suppresses macrophage migration and systemic inflammation**^**(35)**^	**DCA(PDK inhibitor)**
**BMDMs/MoDMs/in vivo**	**LPS /IFNγ**	**itaconate**	**Itaconate exerts anti-inflammatory effects**^**(48, 49, 51)**^	
**MoDMs/(U937/PMA cells)**^**(46)**^**;** **BMDMs/LPS-induced** **model of peritonitis**^**(43)**^	**LPS/ TNFα/IFNγ**^**(46)**^**; LPS**^**(43)**^	**ACLY**	**ACLY exerts pro-inflammatory effects**^**(43, 46)**^	**Radicicol (RAD) /hydroxycitrate (HCA)/SB-204990**^**(46)**^**; BMS 303141 (BMS)**^**(43)**^
**BMDMs**	**LPS**	**Succinate**	**Succinate exerts pro-inflammatory effects**^**(53)**^	
**BMDMs/U937** **/mice with antigen-induced arthritis**	**LPS/IFNγ**	**GPR91**	**GPR91 senses extracellular succinate to enhance IL-1β production**^**(55)**^	**GPR91A1(GPR91 antagonist)**
***Lipid synthesis***				
**BMDMs/mice with injection of LPS**	**LPS**	**SREBP-1a**	**SREBP-1a couples lipogenesis with the NLRP3 inflammasome activation**^**(60)**^	
**BMDMs/PMs/J774A.1 /in vivo**	**LPS**	**FASN**	**Inhibition of FASN suppresses NLRP3 inflammasome activation**^**(61)**^	**C75 and cerulenin(FASN inhibitors)**
**BMDMs**	**IL-4**	**FAS**	**FAS could be contributing to the fueling of FAO, which is essential for M2 activation**^**(37)**^	**C75(FAS inhibitor)**
***Fatty acid oxidation***				
**BMDMs/primary human macrophages/a mouse model of S. pneumoniae lung infection**	**LPS/ATP/nigericin**	**NOX4**	**NOX4-dependent fatty acid** **oxidation promotes NLRP3 inflammasome activation** ^**(63)**^	**GKT137831 and VAS-2870(NOX4 inhibitors)**
***Glutamine metabolism***				
**BMDMs**	**IL-4**	**glutamine**	**Glutamine regulates M2 polarization**^**(40, 71)**^	
**MoDMs/in vivo**	**IL-10**	**Glutamine**	**Inhibition of GS skews macrophages toward an M1-like phenotype and Inhibits tumor metastasis**^**(73)**^	**methionine sulfoximine(GS inhibitor)**
		**Synthetase(GS)**		

### Arginine metabolism

**Fig. 1:**
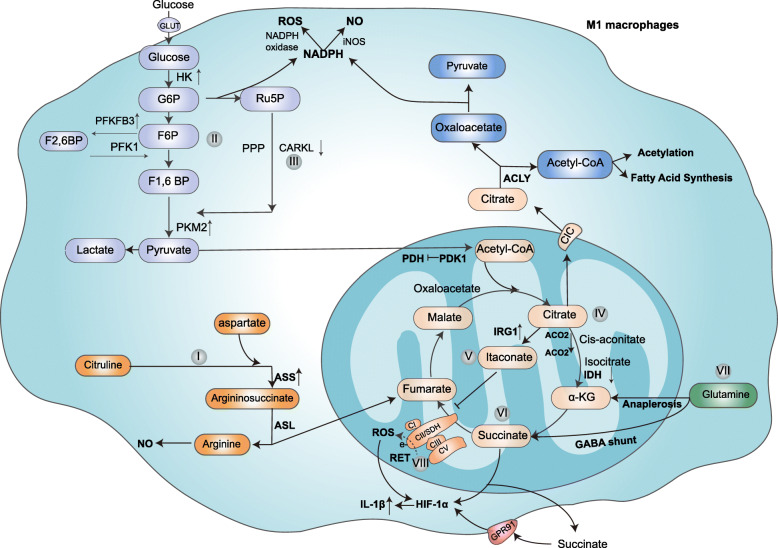
Metabolic characteristics of M1/M [LPS(±IFN-γ)] macrophages. (I) Arginine is metabolized by iNOS to citrulline and NO. Citrulline can be used with aspartate, as catalyzed by Ass1, to generate argininosuccinate, which is further catalyzed by Asl to arginine and fumarate; (II) glycolytic activity is enhanced; (III) PPP activity is increased, generating NADPH for ROS and NO production by NAPDH oxidase and iNOS, respectively; and (IV) citrate accumulates and is then exported into the cytoplasm via CIC and transformed into acetyl-CoA and oxaloacetate via ACLY. Acetyl-CoA is used for FAS and protein acetylation. Oxaloacetate is further metabolized to pyruvate, accompanied by NADPH and NO production. Citrate also generates itaconate via IRG1, thus inhibiting SDH and causing the second break in the TCA cycle. (VI) Succinate accumulates via inhibition of SDH (VII) and derivation from glutamine through anaplerosis via αKG and the GABA shunt pathway. Succinate can stabilize HIF1α, leading to the release of IL-1β. (VIII) The high mitochondrial membrane potential induced by LPS leads to reversal of the normal direction of electron flux through complex III, causing RET in complex I and driving ROS production. Succinate can also be released into the extracellular milieu, where it is sensed by GPR91 to promote inflammatory responses. GLUT, glucose transporter; HK, hexokinase; G6P, glucose-6-phosphate; F6P, fructose 6-phosphate; F1,6BP, fructose-1,6-bisphosphate; F2,6BP, fructose-2,6-bisphosphate; RU5P, ribulose-5-phosphate; PKM2, pyruvate kinase; PFK1, phosphofructokinase1; PDH, pyruvate dehydrogenase; PDK1, pyruvate dehydrogenase kinase 1; iNOS, inducible nitric oxide synthase; MDH, malate dehydrogenase; αKG, α-ketoglutarate; ASS, argininosuccinate synthase; ASL, isocitrate dehydrogenase; RET, reverse electron transport; IRG1, immunoresponsive gene 1; IDH, isocitrate dehydrogenase; ACO2, aconitase 2; CARKL, carbohydrate kinase-like protein; ACLY, ATP-citrate lyase; SDH, succinate dehydrogenase; CIC, citrate carrier

Arginine metabolism is complex due to the expression of multiple enzymes that utilize arginine as a substrate for the synthesis of not only proteins but also NO, urea, polyamines, proline, glutamate, creatine and agmatine [9]. M1 and M2 macrophages have different arginine metabolism characteristics. M1 macrophages express iNOS, which metabolizes L-arginine into NO and L-citrulline (Fig. 1) [7, 9]. The NO produced by activated M1 macrophages exerts antimicrobial effects [10].

In addition, NO and NO-derived reactive nitrogen species can inactivate the mitochondrial electron transport chain (ETC) and prevent M1 repolarization to the M2 phenotype [11]. Similarly, another study showed that NO contributes to ETC impairment and promote protective processes to mitigate NO-induced damage in bone marrow-derived macrophages (BMDMs) stimulated with LPS/IFN-γ【BMDMs (LPS/IFN-γ)】 [12]. Moreover, this group emphasized that NO-mediated suppression of ACO2 causes TCA cycle alteration and can inhibit carbon flux into the TCA cycle through prolyl hydroxylase (PHD) in a hypoxia-inducible factor (HIF1α)-independent manner, thereby promoting glutamine-based anaplerosis [12]. In addition, studies have shown that NO itself has dual regulatory effects on iNOS expression in RAW 264.7 cells stimulated with LPS. When the local concentration of NO is low (usually in the early stages of inflammation), iNOS expression can be increased. However, a high concentration of NO has the opposite effect, downregulating the proinflammatory response of macrophages via expression of iNOS and COX-2. This biphasic activity of NO can facilitate both the initiation of a defense response against pathogenic stimuli and the termination of this response to limit tissue damage [13]. Importantly, citrulline produced by M1 macrophages can be catalyzed by argininosuccinate synthase (Ass1) and argininosuccinate lyase (Asl) to recover arginine and lead to the production of NO (Fig. 1I). The authors noted that during the early stages of NO production, macrophages rely mainly on extracellular arginine to synthesize NO. Later, when exogenous arginine is depleted, cells rely on citrulline to synthesize arginine to sustain optimum NO production in fetal liver-derived macrophages stimulated with LPS + IFN-γ [14]. Collectively, NO can dampen mitochondrial function and the local concentration of NO is critical in determining the pro-inflammatory or anti-inflammatory response in M1 macrophages to maintain the balance between host defense and tissue damage.

**Fig. 2:**
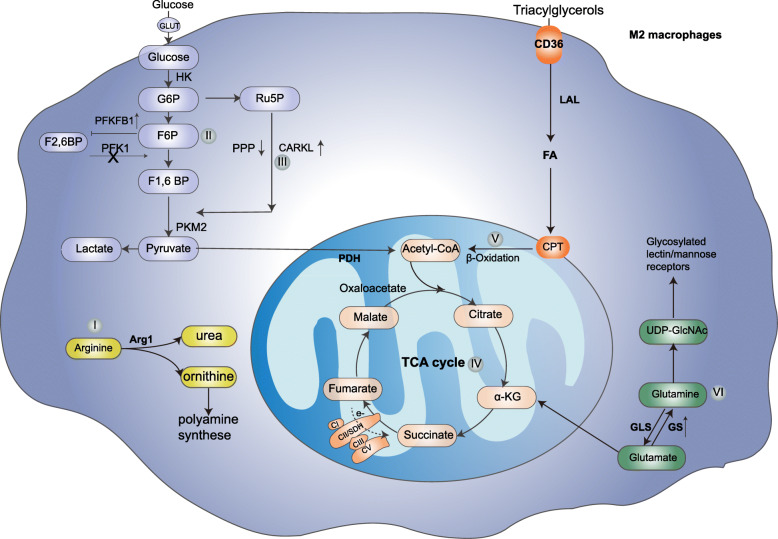
Metabolic characteristics of M2/M [IL-4/IL-10] macrophages. (I) Arginine is metabolized by arginase to urea and ornithine, which participates in the synthesis of polyamines; (II) glycolytic activity is decreased, as M2 may selectively express PFKFB1, which has a low net activity to maintain higher F2,6BP concentrations that can promote PFK1 activity; (III) PPP activity is decreased because of CARKL upregulation; (IV) the TCA cycle is intact; (V) FAO pathway activity is enhanced; and (VI) glutamine metabolism is increased. Glutamine can contribute to replenishment of the TCA cycle and the production of UDP-GlcNAc, which promotes the glycosylation of lectin/mannose receptors—among the most typical M2 polarization markers. In addition, GS is expressed in M2 macrophages and plays a positive role in M2 (IL-10) polarization. GLUT, glucose transporter; HK, hexokinase; G6P, glucose-6-phosphate; F6P, fructose 6-phosphate; F1,6BP, fructose-1,6-bisphosphate; F2,6BP, fructose-2,6-bisphosphate; RU5P, ribulose-5-phosphate; α-KG, α-ketoglutarate; CARKL, carbohydrate kinase-like protein; PKM2, pyruvate kinase; PFK1, phosphofructokinase1; PDH, pyruvate dehydrogenase; CPT, carnitine palmitoyltransferase; FA, fatty acid; LAL, lysosomal acid lipase; GLS, glutaminase; GS, glutamine synthetase; Arg1, arginase 1

In contrast to M1 macrophages, M2 macrophages express Arg, which hydrolyzes arginine to ornithine and urea. Ornithine can further participate in downstream pathways of polyamine and proline synthesis, which are important for cell proliferation and tissue repair (Fig. 2I) [9].

### Glycolysis

Glycolysis is the metabolic pathway that converts glucose into pyruvate, which is controlled by various glycolytic enzymes (Fig. 1). Glycolytic metabolism is an inefficient pathway in terms of ATP production, as it generates only two ATP molecules per unit of glucose. Moreover, it provides intermediate products for the synthesis of other macromolecules, such as nucleotides and amino acids [15]. Tumor cells preferentially use glycolysis to produce lactate under normoxic conditions; this phenomenon is called “aerobic glycolysis” or the “Warburg effect” and was discovered by Warburg et al. in the early twentieth century [16]. Subsequently, increased aerobic glycolysis was observed in proinflammatory macrophages in many other studies [17–20]. Van den et al. used extracellular flux analysis to determine that LPS-induced BMDMs exhibit enhanced glycolytic metabolism [21]. Increasing evidence has shown that glycolytic activity is critical for macrophage function. It has been demonstrated that overexpression of glucose transporter 1 (GLUT1), which is a primary rate-limiting glucose transporter, can enhance glucose uptake and metabolism, thereby modulating the macrophage inflammatory response by elevating the secretion of inflammatory mediators in LPS-stimulated RAW264.7 cells as well as in adipose and liver tissues of both lean and diet-induced obese rats. Notably, pharmacological inhibition of glycolysis with 1 mM 2-deoxy-D-glucose (2-DG) reversed these phenomena (Fig. 1II) [22].

Moreover, Pavlou et al. demonstrated that glycolysis in peritoneal macrophages elicited by Brewer thioglycollate broth injection was highly correlated with phagocytic activity because inhibiting glycolysis with 2-DG (≥5 mM) or promoting glycolysis with oligomycin reduced or increased phagocytosis, respectively [23]. Strikingly, the impact of glycolysis on macrophage functions must be interpreted with caution, as the glycolysis inhibitor 2-DG has an off-target effect. This effect is discussed in detail in the following sections. Further study assessing the role of glycolysis in M1 macrophages by inhibiting glycolysis via galactose or glucose depletion is needed. Although glycolysis produces only two molecules of ATP, far less than the amount generated by OXPHOS (36 ATP molecules from a single molecule of glucose), glycolysis can be activated more rapidly to match the immune responses of macrophages [24, 25]. Collectively, glycolysis may promote the innate immune function of macrophages by increasing the secretion of inflammatory cytokines and enhancing phagocytic activity.

#### Multiple roles of glycolytic enzymes

The recent literature show that glycolytic enzymes play important roles in macrophage functions. Hexokinase (HK), which regulates the first step in glucose metabolism, catalyzes glucose to glucose-6-phosphate (G6P) [26]. Moon JS et al. demonstrated that mammalian target of rapamycin complex 1 (mTORC1)-induced HK1-dependent glycolysis can promote NLRP3 inflammasome activation, which regulates interleukin (IL)-1β and IL-18 processing and secretion in peritoneal macrophages in response to LPS and ATP stimulation [27]. Importantly, a more recent study showed that HK can participate in metabolism by acting as a pattern recognition receptor that recognizes peptidoglycan, which leads to NLRP3 inflammasome activation and IL-1β production in LPS-primed BMDMs (Fig. 1II) [28].

It has been demonstrated that a switch in the expression of 6-phosphofructo-2-kinase/fructose-2,6-bisphosphatase3 (PFK2/PFKFB3) from liver-type PFK2 (L-PFK2) to the more active ubiquitous PFK2 isoenzyme encoded by the PFKFB3 gene increases the fructose-2,6-bisphosphate (F2,6BP) concentration and promotes glycolytic flux [20]. Moreover, Jiang et al. showed that type I IFN, which is triggered by viral infection, can preferentially upregulate the glycolytic activator PFKFB3 in mouse TMPMs and BMDMs, thus promoting the engulfment and removal of virus-infected cells (Fig. 1II) [29].

The expression of the phosphofructokinase muscle (PFK-M) isoform can enhance macrophage glycolysis, driving host defenses against *Mycobacterium tuberculosis* [30].

Glyceraldehyde-3-phosphate dehydrogenase (GAPDH) is an energy metabolism-related enzyme that generates NADH during glycolysis. In resting BMDMs, GAPDH binds to tumor necrosis factor-α (TNFα) mRNA and suppresses its translation. Upon LPS stimulation [BMDMs (LPS)], GAPDH undergoes malonylation at lysine 213, leading to its dissociation from TNFα mRNA and thus promoting TNFα translation [31].

In glycolysis, pyruvate kinase (PK) is the rate-limiting enzyme that converts phosphoenolpyruvic acid (PEP) to pyruvate. Pyruvate kinase M2 isoform (PKM2) exists primarily as an enzymatically inactive monomer or dimer but can also exist in a highly active tetrameric form (Fig. 1II). Previous studies have shown that LPS-induced PKM2 forms a complex with HIF1α, which can directly bind to the promoter of not only IL-1β, promoting its production, but also a range of other HIF1α-dependent genes in BMDMs (LPS) [32]. Another study showed that PKM2-mediated glycolysis promotes inflammasome activation by modulating EIF2AK2 phosphorylation in LPS-primed BMDMs, consequently promoting the release of IL-1β, IL-18 and HMGB1. Pharmacological inhibition of the PKM2-EIF2AK2 pathway protects mice from lethal endotoxemia and polymicrobial sepsis [33]. Collectively, these findings reveal a novel PKM2-dependent mechanism for the metabolic control of inflammation.

Pyruvate dehydrogenase kinase 1 (PDK1), a key regulatory enzyme in glucose metabolism, plays an important role in the activation of macrophages. PDK1 induces the inhibitory phosphorylation of pyruvate dehydrogenase (PDH) complex components, thereby preventing pyruvate from producing acetyl-coenzyme A (CoA) and entering the TCA cycle. Consistent with this, a previous study showed that PDK1 knockdown diminished LPS-induced M1 macrophage activation by attenuating the expression of TNFα and IL-6 and enhancing mitochondrial respiration during the early activation of IL-4-stimulated macrophages [34]. Moreover, studies have shown that in addition to playing a role in macrophage polarization, PDK1 promotes glycolysis in RAW 264.7 cells and murine TMPMs under hypoxic conditions and then promotes macrophage migration [35].

#### Glycolysis and M2 macrophages

The metabolic signature of M2 macrophages is quite different from that of M1 macrophages. M2 macrophages do not exhibit increased glycolysis. Previous studies have shown that the trend in glycolytic enhancement after activation of peritoneal macrophages (IL-4/IL-13 and IL-10) is not significant because it may result in selective expression of L-PFK2, which is encoded by the 6-phosphofructo-2-kinase/fructose-2, 6-biphosphatase 1 (PFKFB1) gene and has low net activity to maintain higher F2,6-BP concentrations, thereby decreasing glycolytic flux (Fig. 2II) [20].

However, several recent studies have documented that glycolysis plays a role in M2 activation. Glucose oxidation has been demonstrated to be required for the early differentiation of M2 macrophages, as the glycolytic inhibitor 2-DG (1 mM) can attenuate enhanced mitochondrial respiration and impair the activation of early-stage IL-4-stimulated BMDMs, as assessed via the detection of phenotypic markers such as Arg1, YM-1, FIZZ-1 and MRC1 [34]. Similar findings were demonstrated by Bossche et al. [11], who demonstrated that 2-DG (1 mM)-mediated blockade of glycolytic flux suppressed mitochondrial respiration and impaired BMDMs (IL-4)-associated expression of the Arg1, Cdh1, Chi3l3, Mrc1 and Retnla genes. Covarrubias et al. showed that, mechanistically, glycolysis is enhanced in BMDMs (IL-4) through AKT and mTORC1 and that 2-DG (0.5 M) reduces the IL-4-mediated induction of specific M2 genes, including Arg1, Mgl2 and Retnla [36]. A subsequent study showed that mTORC2 signaling upstream of IRF4 expression is a critical mechanism in glucose metabolism that is essential for BMDMs (IL-4) activation, as the expression of the M2 activation markers resistin-like alpha (RELMa) and programmed cell death ligand 2 (PD-L2) was markedly inhibited by 2-DG (10 mM). Similar results were obtained in cells cultured in glucose-free medium [37].

However, the most recent studies have shown that depletion of glucose or substitution of glucose with galactose, which similarly suppresses glycolysis but does not affect OXPHOS, does not affect BMDMs (IL-4) activation, as evaluated via the detection of RELMa and PD-L2. In addition, 2-DG may have additional off-target effects at higher doses (10 mM), as it not only impairs glycolysis and OXPHOS but also reduces the intracellular ATP concentration and JAK-signal transducer and activator of transcription 6 (STAT6) signaling, which in turn affects M2 differentiation [38]. Interestingly, inhibition of OXPHOS with the mitochondrial ATP synthase inhibitor oligomycin has been reported to render macrophages dependent on glycolysis for ATP production. This finding is consistent with data described previously [11]. This pattern demonstrated that glycolysis is not mandatory for M2 activation if OXPHOS is intact, but becomes required if OXPHOS is compromised. Intriguingly, reports have demonstrated that blocking mitochondrial pyruvate transport with UK-5099 [37] or genetic depletion of PDK1 [34] can impair M2 activation. These observations may suggest that UK-5099 and PDK1 regulate M2 macrophage activation by a mechanism unrelated to their function of fueling pyruvate synthesis through glycolysis. Collectively, an increasing amount of evidence suggests that glycolysis is essential for M2 activation. However, the most recent studies have shown that glycolysis does not affect M2 activation. Therefore, the specific role of glycolysis in M2 needs further delineation and remains a focus of future studies.

### The pentose phosphate pathway (PPP)

The PPP, which branches from glycolysis at the first committed step of glucose metabolism. The glycolytic intermediate metabolite G6P can be dehydrogenated to produce 6-phosphogluconic acid (6PG). In turn, 6PG can be dehydrogenated by 6-phosphogluconate dehydrogenase (6PGD) to generate ribulose-5-phosphate (Ru5P), a precursor of nucleotides and amino acids that are necessary for cell growth and proliferation. Additionally, this pathway constitutes a major source of NADPH, which is used for reactive oxygen species (ROS) production by NADPH oxidase and is also required for both the generation of the antioxidant glutathione and FAS (Fig 1III) [25, 39].

Research has reveled that LPS-stimulated BMDMs and TMPMs exhibit increased PPP flux [40, 41]. Carbohydrate kinase-like protein (CARKL), a sedoheptulose kinase, can limit PPP. Haschemi et al. discovered that upon LPS-induced macrophage activation, a highly significant reduction in CARKL expression, which can induce the production of proinflammatory factors (Fig. 1III) [41]. In contrast to the M1-like activation of macrophages, TMPMs and BMDMs activation by IL-4 results in upregulation of CARKL, which contributes to suppression of the PPP (Fig. 2III) [41].

### TCA cycle

The TCA cycle is driven by the reaction acetyl coenzyme A (acetyl-CoA), generated from glucose-derived pyruvate, fatty acids or α-ketoglutarate (αKG) derived from glutamate, combines with oxaloacetate to form citrate, which is then decarboxylated and oxidized resulting in malate, from which the starting oxaloacetate is regenerated, completing the cycle. In a series of enzymatic reactions the TCA cycle generates two major products NADH and FADH2, which can transfer electrons to the ETC to support oxidative phosphorylation and highly efficient ATP generation, which requires oxygen and it is known as OXPHOS [42] (Fig. 1).

In M2 macrophages, the TCA cycle proceeds in conjunction with OXPHOS to generate ATP. However, the metabolic signature of M1 macrophages is quite different from that of M2 macrophages. In M1 macrophages, the TCA cycle has been shown to be broken in two places—after citrate and after succinate. These breaks result in the accumulation of certain metabolites, for example, citrate, itaconate and succinate [40].

#### Citrate

Normally, citrate is converted to isocitrate and then to αKG through the enzymatic activity of isocitrate dehydrogenase (IDH) (Fig. 1IV). A recent study demonstrated that disruption of the TCA cycle and accumulation of citrate are likely due to transcriptional downregulation of IDH in BMDMs (IL-4/IFN-γ) (Fig. 1IV) [40]. Importantly, the most recent study demonstrated that NO-mediated suppression of mitochondrial aconitase 2 (ACO2) might be responsible for disruption of the TCA cycle (Fig. 1IV) [12]. Interestingly, another recent study reported that citrate accumulation is probably dependent on pyruvate. The researchers observed a global increase in citrate levels in BMDMs in response to LPS stimulation for 2 h, while treatment with the mitochondrial pyruvate carrier 1 (MPC-1) inhibitor UK5099 reduced the cellular concentrations of citrate and aconitate [43].

The citrate accumulated in the mitochondria of M1 macrophages is exported into the cytoplasm via the mitochondrial citrate carrier (CIC), which is encoded by the SLC25A1 gene (Fig. 1IV). In addition, in macrophages (U937/PMA cells) stimulated by proinflammatory factors such as LPS, TNFα and IFN-γ, the expression of CIC is markedly increased, and the production of inflammatory mediators is enhanced [44, 45].

Indeed, cytoplasmic citrate plays a crucial role in sustaining the macrophages inflammatory response. Citrate is transformed into acetyl-CoA and oxaloacetate via ATP-citrate lyase (ACLY), which is increased in inflammatory macrophages and is essential for the production of prostaglandin E2 (PGE2), NO and ROS mediators [46]. Inhibition of either CIC or ACLY can reduce the production of prostaglandin as well as NO and ROS, which contribute to inflammatory macrophage responses [44–46]. Oxaloacetate, another product of citrate metabolism, is further metabolized to pyruvate. This process is accompanied by the production of NADPH, which is used by NADPH oxidase or iNOS to produce ROS or NO, respectively (Fig. 1IV) [46].

Additionally, citrate plays a major role in controlling cell metabolism and regulating protein functions. Research has shown that LPS-stimulated macrophages can activate the adaptor proteins MyD88 and TRIF, resulting in the activation of ACLY, through which acetyl-CoA can promote protein acetylation and the expression of inflammatory genes [43]. As stated above, citrate plays an important role in proinflammatory macrophage responses.

#### Itaconate

Itaconate is synthesized through decarboxylation of cis-aconitate, which is derived from citrate, by immunoresponsive gene 1 (IRG1) in the mitochondrial matrix (Fig. 1IV) [47].

In LPS-stimulated macrophages, there were strong upregulation of both the IRG1 transcript and itaconic acid synthesis (Fig. 1V) [12, 40]. Furthermore, itaconic acid production promotes the antibacterial effects of macrophages [47]. In addition, itaconate may play an important role in the metabolism and function of macrophages.

Pretreatment of mouse BMDMs with physiologically relevant doses of dimethyl itaconate (DI), a membrane-permeable nonionic form of itaconate, suppresses iNOS expression; IL-12p70, IL-6 and ROS secretion; and inflammasome activation in vivo and in vitro. These activities indicate that itaconic acid has anti-inflammatory effects. Researchers have shown that, mechanistically, the anti-inflammatory effects of itaconate in BMDMs are likely due to both inhibition of succinate dehydrogenase (SDH) and regulation of the levels of succinate, which can control ETC flux through ROS production and regulation of the inflammatory response (Fig. 1VIII) [48]. A recent study showed that itaconate also exerts its anti-inflammatory effects via activation of the anti-inflammatory transcription factor Nrf2 (also called NFE2L2). Itaconate is required for LPS-induced Nrf2 activation in mouse and human macrophages by alkylating crucial cysteine residues of KEAP1, enabling Nrf2 to increase the expression of downstream genes with antioxidant and anti-inflammatory capacities [49, 50].

Bambouskova et al. demonstrated that due to their electrophilic properties, itaconate and its derivatives react with glutathione and induce both Nrf2-dependent and Nrf2-independent responses; moreover, they showed that the electrophilic stress caused by itaconate can regulate the IL-6 response, which is a regulatory secondary transcriptional response to Toll-like receptor (TLR) stimulation, via the IκBζ-ATF3 inflammatory axis [51].

Strikingly, the most recent studies have shown that 4-octyl itaconate (4-OI, a cell-permeable itaconate derivative) alkylates cysteine residues of GAPDH and downregulates aerobic glycolysis, exerting anti-inflammatory effects in RAW264.7 macrophages and BMDMs (LPS) [52]. Collectively, all of these studies point towards itaconate exhibits an anti-inflammatory effect, which may limit damage to macrophages under proinflammatory conditions.

#### Succinate

Succinate has been demonstrated to accumulate in LPS-stimulated macrophages (Fig. 1VI) [53]. There may be at least two important reasons for this phenomenon. First, the conversion of succinate to fumarate in M1 macrophages is not efficient, probably due to SDH inhibition [40]. Second, succinate could accumulate by a process related to glutamine anaplerosis via αKG and the GABA shunt pathway. Indeed, microarray screening gene expression analysis and metabolomic studies have revealed that LPS stimulation can significantly increase the expression of a glutamine transporter (SLC3A2) and GABA transporters (SLC6A13 and SLC6A12), and the production of GABA. Researchers have also analyzed the metabolism of ^13^C5, ^15^N2-labeled glutamine and measured the increase in ^13^C4-succinate abundance in BMDMs treated with LPS for 24 h. The elevation of succinate derived from glutamine was found to be largely due to anaplerosis via αKG, although a proportion was derived from the GABA shunt pathway (Fig. 1VII) [53].

Accumulated succinate can then be transported from mitochondria to the cytoplasm by the dicarboxylate carrier DIC (SLC25A10) and exert an array of effects [54].

Within the cytosol, succinate has been shown to inhibit the activity of PHD and thereby stabilize HIF1α, leading to the release of proinflammatory cytokines such as IL-1β under normoxic conditions in BMDMs (LPS) (Fig. 1VI) [53].

Moreover, succinate can increase the succinylation of several proteins, such as malate dehydrogenase, GAPDH, glutamate carrier 1, and lactate dehydrogenase (LDH). Further research is needed to define the consequences of the succinylation of these proteins [53].

In addition, when BMDMs are stimulated by inflammatory signals (LPS + IFN-γ), the expression of SUCNR1/GPR91 (the succinate receptor) is upregulated, and succinate is released into the extracellular matrix. Then, extracellular succinate binds to GPR91, which functions in both an autocrine and a paracrine manner to promote the production of IL-1β, fueling tissue inflammation (Fig. 1VI) [55].

Interestingly, a recent study showed an increase in succinate in the brain, kidneys, liver, and heart of a mouse model of ischemia-reperfusion (IR). The authors demonstrated that succinate accumulation during ischemia might result from SDH reversal. Upon reperfusion, succinate is rapidly reoxidized by SDH, driving ROS generation by RET in mitochondrial complex I. Therefore, decreasing ischemic succinate accumulation by pharmacological inhibition is sufficient to ameliorate IR injury in murine models of heart attack and stroke [56]. Collectively, these findings provide new insight into succinate accumulation. However, whether SDH reversal also occurs during macrophage activation warrants further study.

#### Replenishment of the TCA cycle

Disruption of the TCA cycle in M1 macrophages requires a series of reactions to supply intermediates that fuel the TCA cycle. Succinate derived from glutamine is produced largely by anaplerosis via αKG and by the GABA shunt pathway, as mentioned previously. In addition, an increase in the aspartate-arginosuccinate shunt in M1 macrophages provides the metabolites fumarate and malate for the disrupted TCA cycle (Fig. 1I) [40]. Mechanistically, accumulated intracellular citrulline metabolized by iNOS in M1 macrophages can be used with aspartate via a process catalyzed by Ass1 to generate argininosuccinate. In turn, argininosuccinate is further catalyzed by Asl to generate arginine and fumarate, the latter of which replenishes the TCA cycle [14, 40]. This replenishment is an important metabolic adjustment that provides intermediates immediately downstream of the break in the TCA cycle.

### Lipid metabolism

The FAS pathway is an essential cellular process, which is necessary for membrane biosynthesis and energy storage. Generally, citrate derived from the TCA cycle is exported from mitochondria into the cytosol and transformed into acetyl-CoA and oxaloacetate via ACLY. Acetyl-CoA is then converted to malonylCoA by acetyl-CoA carboxylase (ACC). Subsequently, FA synthase (FASN) elongates the nascent fatty acid chain until generates palmitate, the initial product of FAs. Furthermore, fatty acids may be elongated and desaturated to produce molecules of various lengths and degrees of saturation. In addition, intracellular fatty acids may be used for the synthesis of triacylglycerides for energy storage, glycerophospholipids, cardiolipins and sphingolipids for membrane synthesis, and eicosanoids for signaling processes [57].

The fatty acid oxidation (FAO) pathway is important in cellular homeostasis as it is involved in the process of energy production. The initial step of FAO is the CoA group added to the FA by fatty acyl CoA synthetase to generate fatty acid acyl-CoA, which is then conjugated to carnitine via carnitine palmitoyltransferase I (CPT1) in the mitochondrial outer membrane and transported to the mitochondrial matrix. Subsequently, the long-chain fatty acyl carnitine is then converted back to fatty acyl CoA with the help of carnitine palmitoyltransferase II (CPT2), which produces acetyl-CoA, NADH and FADH2; these molecules are further used in the TCA cycle and the ETC to generate ATP [58].

There are increasing reports showed that altered lipid metabolism has been associated with immune responses. Feingold et al. demonstrated that in LPS-treated RAW 264.7 cells, the conversion of glucose to lipids is increased, and FA uptake is enhanced [59].

Strikingly, recent studies have shown crosstalk between lipid synthesis and the macrophage proinflammatory response. Im et al. showed that the expression of SREBP-1α, which activates not only de novo lipogenesis but also the Nlrp1α gene, a core inflammasome component that promotes the secretion of proinflammatory cytokines, is increased in LPS-activated BMDMs [60]. Similarly, another study showed that during macrophage activation, mitochondrial uncoupling protein-2 (UCP2) increases lipid synthesis by increasing the level of FASN, a key regulator of FAS. Moreover, this study observed that UCP2 can induce NLRP3-mediated caspase-1 activation to promote IL-1β and IL-18 secretion in BMDMs (LPS) [61]. In addition, exogenous saturated fatty acids can activate the inflammasome by increasing the level of saturated phosphatidylcholine (PC), which is correlated with a loss of membrane fluidity and promotes the disruption of the plasma membrane Na, K-ATPase, causing K+ efflux [62]. Interestingly, recent studies have suggested that FAO may contribute to inflammasome activation. A previous report revealed that NADPH oxidase 4 (NOX4), which is a source of cellular superoxide anions, induces FAO via CPT1A during NLRP3 inflammasome activation and contributes to the phenotype of inflammatory macrophages [63]. These results suggest the novel idea that LPS-activated macrophages require not only FAS but also FAO for NLRP3 activation.

In addition, a recent study demonstrated that during macrophage colony-stimulating factor (M-CSF)-stimulated differentiation of human monocytes into macrophages, the sterol regulatory element-binding protein (SREBP) gene, which can promote FAS, is upregulated. The researchers further noted that the synthesis of fatty acids promotes the plasticity of pseudopods and thus promotes macrophage phagocytosis because the inhibition of FAS prevents phagocytosis [64].

In contrast to M1 macrophages, studies have shown that M2 macrophages exhibit enhanced FAO. Previous studies have demonstrated that pharmacological inhibition of FAO by the mitochondrial CPT1 inhibitor etomoxir (50 μM or 200 μM) dramatically inhibits IL4-stimulated M2 activation, as detected by reductions in arginase activity and dectin-1 and mannose receptor expression or the expression of CD301, CD206 and RELMα, respectively [65, 66]. Moreover, fatty acids in BMDMs (IL-4) are derived through uptake of triacylglycerol (TAG) substrates via CD36 and lipolysis by lysosomal acid lipase (LAL) (Fig. 2V). Notably, researchers have also shown that M2 macrophages can utilize endogenous TAGs generated via de novo FAS [66]. Huang et al. also demonstrated that inhibiting FAS prevented IL-4-induced M2 activation. These effects indicated that FAS may be essential for M2 activation [37].

However, follow-up studies have shown that inhibition of FAO with the CPT1 inhibitor etomoxir (50 μM or 10 μM) is dispensable for IL4-induced activation of mouse and human macrophages, as evidenced by evaluations of Arg1, Cdh1, Chi3l3, Mrc1 and Retnla mRNA levels and CD71, CD206 and CD301 or CCL18 and Mrc1 mRNA levels, respectively [11, 67]. Importantly, another study using a genetic model showed that in macrophages lacking CPT2, in which FAO cannot occur, etomoxir (200 μM) equally inhibited the M2 polarization markers Arg1, Mgl2 and Retnla [68]. These results may be explained by the off-target effects of etomoxir, which might react with a broad range of intracellular nucleophiles. Alternatively, CPT1, as the presumed target of etomoxir, might participate in some functions independent of long-chain FAO.

Strikingly, a recent study using genetic and pharmacological models showed that FAO is not essential during macrophage polarization—genetic deletion of Cpt1a had little impact on macrophage polarization in BMDMs (IL-4), as determined by the population of CD206+/CD71+ cells. A similar result was observed for genetic deletion of Cpt2. Interestingly, high concentrations of etomoxir can disrupt IL-4-stimulated macrophage polarization in the absence of Cpt1a or Cpt2 expression. These results suggest that the effect of excess etomoxir on IL-4-stimulated macrophage polarization is independent of CPT activity. Indeed, the authors explained that etomoxir has no effect on the IL-4 response at concentrations ranging from 10 mM to 100 mM, and significant effects on macrophage phenotypes were observed only at concentrations > 100 mM. Moreover, researchers have demonstrated multiple off-target effects of etomoxir and shown that depletion of coenzyme A by etomoxir blocks BMDMs (IL-4) differentiation [69].

Collectively, mounting evidence has revealed that FAO is not essential during M2 activation, and FAS may contribute to M2 activation by fueling OXPHOS [37, 66]. However, extensive studies assessing this possibility and the underlying mechanism are still warranted.

### Glutamine metabolism

Glutamine in cells is converted to glutamate by glutaminase (GLS). The next step glutamate is converted to a-KG to replenish the TCA cycle. Moreover, glutamine also contributed to produce glutathione, serving as a precursor to nucleotides and lipid synthesis via reductive carboxylation [70].

As mentioned earlier, LPS-stimulated BMDMs cause the accumulation of succinate, which can be derived from glutamine through the GABA shunt pathway and from αKG through anaplerosis, and can then stabilize HIF1α and promote the macrophage inflammatory response [53].

Interestingly, other studies have recently reported that glutamine metabolism plays an important role in M2 macrophages but not M1 macrophages. These studies demonstrated that transient deprivation of glutamine in BMDMs does not affect M1 polarization (induced by LPS + IFN-γ), as measured by Nos2 expression, but significantly inhibits the expression of M2 (IL-4) markers, as detected by CD206, CD301, and Relma expression, and downregulates the transcriptional signature of TCA cycle activity. Notably, researchers showed that one-third of all carbons in the TCA metabolites of M2 macrophages originate from glutamine. In addition, high levels of UDP-GlcNAc have been observed in M2 macrophages. By tracing the fate of ^15^N-glutamine in M2 macrophages to the production of UDP-GlcNAc, researchers found that more than half of the nitrogen in UDP-GlcNAc is derived from glutamine [40]. These findings indicate that glutamine-related metabolism and the UDP-GlcNAc pathway play an important role in M2 polarization [40]. Similar findings were reported in a study conducted by Liu et al., which demonstrated that IL-4-stimulated BMDMs show a higher oxygen consumption rate (OCR) and spare respiratory capacity (SRC) than unstimulated BMDMs but that the SRC is not increased under glutamine-deprived conditions. Consistent with these findings, the expression of the M2 markers Arg1, Mrc1, Ym1 and Retnla was found to be increased in IL-4-treated macrophages but decreased under glutamine-deprived conditions [71].

Another study conducted by Nelson et al. revealed that PPARγ, which regulates FAO, plays an important role in glutamine oxidation, as thioglycollate-induced peritoneal macrophages lacking PPARγ cannot effectively respirate using glutamine as fuel [72]. Finally, another study showed that glutamine synthetase (GS), the enzyme that synthesizes glutamine from glutamate, is important for M2 activation. Pharmacological inhibition of GS polarizes IL-10-stimulated primary human monocyte-derived macrophages (MoDMs) toward the M1-like phenotype, as indicated by the strong appearance and upregulation of CD80 accompanied by the disappearance of CD163 as well as several differentially expressed genes. This effect is mediated at least partially via HIF1α, which can possibly be stabilized by succinate accumulation (Fig. 2VI) [73]. Collectively, these findings suggest that glutamine metabolism plays an important role in macrophage polarization.

## Metabolic profiles of TAMs

The characteristics of macrophage metabolism described above focus mostly on murine BMDMs in vitro. Although M1 and M2 macrophages have distinct phenotypic and metabolic characteristics, the situation in vivo is more complex. TAMs reprogram their metabolism in response to environmental cues to mediate their function in the tumor microenvironment (TME). In general, TAMs exhibit enhanced glycolysis, FAS, FAO and altered glutamate metabolism while maintaining a tumor-promoting function. Liu et al. demonstrated that glycolysis activity was enhanced in tumor extract-stimulated BMDMs (TES-TAMs) and that HK2 was upregulated in both TES-TAMs and TAMs isolated from a breast cancer mouse model [74]. These results were consistent with findings in pancreatic ductal adenocarcinoma (PDAC) [75] or non-medullary thyroid carcinoma [76] induced macrophages. Interestingly, another study showed that under hypoxic conditions, TAMs exhibit upregulation of regulated in development and DNA damage response 1 (REDD1) an mTORC1 inhibitor, which subsequently inhibits the glycolytic shift toward oxidative metabolism. Thus, increasing glucose availability in the perivascular space causes endothelial hyperactivation, which leads to neoangiogenesis and metastasis [77]. These findings indicate that the TME exerts a profound influence on the phenotype and function of TAMs.

Moreover, a number of studies have shown that lipids are a crucial factor in regulating the function of TAMs. A study conducted by Xiang et al. revealed that lipid accumulation in TAMs resulted from monoacylglycerol lipase (MGLL) deficiency, which can promote CB2/TLR4-dependent macrophage activation and further suppress the function of tumor-associated CD8+ T cells and the progression of multiple cancers [78]. In addition, a growing body of research suggests that activation of peroxisome proliferator-activated receptor β/δ (PPARβ/δ) and FAO contributes to the pro-tumorigenic roles of TAMs. Schumann et al. demonstrated that the vast majority of direct PPARβ/δ target genes are upregulated in TAMs, contributing to the protumorigenic polarization of ovarian carcinoma TAMs [79]. Park et al. showed that PPARβ/δ activation in tumor myeloid cells enhances tumor angiogenesis and malignant cell invasion in an IL-10-dependent manner. This effect was initiated by M-CSF from cancer cells, which activated PPARβ/δ in tumor myeloid cells via increased levels of endogenous FASN [80]. The latest research demonstrated that TAMs express elevated levels of CD36, accumulate lipids, and use FAO instead of glycolysis for energy. Inhibition of lipid uptake or FAO in macrophages can block the generation of TAMs and abolish their protumor functions in vitro and in vivo [81]. Moreover, another study showed that receptor-interacting protein kinase 3 (RIPK3) deficiency promoted the infiltration and M2 differentiation of TAMs via metabolic reprogramming of fatty acids in the TME and that FAO blockade reversed the immunosuppressive activity of TAMs and suppressed HCC tumorigenesis [82]. These findings indicate that lipid metabolism plays an important role in mediating TAM function.

The effects of glutamate metabolism in TAMs on their functional phenotype have rarely been investigated. Choi et al. demonstrated that TAMs isolated from resected glioblastoma tissue or exposed to glioblastoma cells exhibit enhanced expression of several genes related to glutamate transport and metabolism [83]. The exact mechanism is unknown, further studies are needed.

In addition, tumor cells can shape TAMs to exert an immunosuppressive phenotype that limits immune responses. Malignant cancer cells exhibit a high rate of aerobic glycolysis, which produces lactic acid in the TME [84]. Research has shown that lactic acid has an important role in the induction of M2-like polarization of TAMs [85, 86]. Mechanistically, the author demonstrated that lactate can stabilize HIF1α and induce vascular endothelial growth factor (VEGF), Arg1 and other M2-associated genes in TAMs. In summary, TAMs promote tumor growth through altered immunomodulation and their phenotype can be altered in response to local environmental cues. Thus, the results that we obtained for specific cytokines in vitro can be used only as a reference, and specific metabolic changes and functional states need further verification in specific models in vivo.

## Immunometabolism editing as a target for cancer therapy

As stated above, macrophage-specific metabolic alterations are a major driving force mediating macrophage function. Moreover, tumor cells also have remarkable metabolic requirements in contrast to normal differentiated cells. Cancer cells exhibit a high rate of metabolic activity, such as glycolysis and glutamine metabolism [84]. Based on these observations, the development of treatments that target key metabolic enzymes could have important clinical benefits. Next we will summarize several potential drugs for cancer treatment (Table 3).

**Table 3:** Agents targeting cell metabolism for cancer treatment

Agent(s)	Target(s)	Effect(s)	Stage of development	Study number(s)
**CB-1158**	**Arginase 1**	**Inhibits Arginase**	**Phase I/II**	**NCT02903914**
			**Phase I**	**NCT03910530**
			**Phase I/II**	**NCT03314935**
			**Phase I/II**	**NCT03837509**
			**Phase I/II**	**NCT03361228**
**2DG**	**Hexokinase**	**Inhibits glycolysis**	**Phase I(completed)**	**NCT00096707**
**3PO/PFK15**	**PFKFB3**	**Inhibits glycolysis**	**Preclinical**^**(97-100)**^	
**Shikonin**	**PKM2**	**Inhibits PKM2**	**Preclinical**^**(103-105)**^	
**DCA**	**PDK1**	**Inhibits PDK**	**Phase II**	**NCT01386632**
			**Phase I**	**NCT01111097**
			**Phase II**	**NCT00540176**
**GKT137831**	**NOX4**	**Inhibits NOX4**	**Phase II**	**NCT03226067**
**CB839**	**GLS**	**Inhibits glutamine metabolism**	**Phase I/II**	**NCT03875313**
			**Phase I/II**	**NCT02861300**
			**Phase II**	**NCT03163667**
			**Others**	**Others**
**PX-478**	**HIF-1α**	**Inhibits HIF-1α**	**Phase I**	**NCT00522652**
**EZN-2968**	**HIF-1α**	**Inhibits HIF-1α**	**Phase I**	**NCT01120288**
**Temsirolimus/ Everolimus**	**mTOR**	**Inhibits mTOR**	**FDA approved**	**>100**
**Ridaforolimus**	**mTOR**	**Inhibits mTOR**	**Phase II**	**NCT00086125**
			**Phase II**	**NCT00122343**
			**Phase II**	**NCT00739830**
			**Phase III**	**NCT00538239**
			**Phase II**	**NCT00093080**
				**(Completed)**
**Perifosine**	**AKT**	**Inhibits AKT**	**Phase I**	**NCT02238496**

Previous studies have shown that aminoguanidine, an inhibitor of iNOS, can ameliorate experimental autoimmune encephalomyelitis in SJL mice [87]. Moreover, another inhibitor of iNOS, GW274150, can reduce experimental renal ischemia/reperfusion injury and prevent organ injury associated with inflammation in a model of lung injury [88–90]. However, to our knowledge there are no iNOS inhibitors used in antitumor therapy in the clinic.

It was demonstrated that inhibiting Arg1 with CB-1158 blunted myeloid cell-mediated immune evasion and reduced tumor growth [91]. CB-1158 is currently in clinical trials as a single agent or in combination with checkpoint blockade in multiple types of cancer (NCT02903914, NCT03910530, NCT03314935, NCT03837509, NCT03361228).

Blocking glycolysis with 2-DG results in impaired M2 macrophage polarization in vivo and in vitro [92]. 2DG can efficiently slow cell growth and facilitate apoptosis in specific cancers, and it may be combined with other therapeutic agents or radiotherapy to elicit a synergistic anticancer effect [93]. A phase I clinical trial which completed assessed the effects of 2DG used alone or combined with docetaxel against advanced solid tumors. The authors concluded that the combination of 2DG and docetaxel appears to be feasible [94].

Accumulating studies have suggested that PFKFB3 can promote the oncogenesis, proliferation and survival of cancer cells [95]. Two inhibitors of PFKFB3, 3PO and PFK15, have shown antitumor activity in several different solid tumors [96–99]. In light of this evidence, it is clear that targeting PFKFB3 could have important clinical benefits for cancer therapy. However, until now, neither 3PO nor PFK15 has undergone clinical trials.

Pyruvate kinase M2 (PKM2) catalyzes the last step in glycolysis, a key process of tumor metabolism. We have previously described that PKM2 is involved in the proinflammatory activities of M1 macrophages. Moreover, PKM2 has been reported to be associated with tumor progression and promotes the Warburg effect in cancer cells [100, 101]. A clinical trial evaluating PKM2 expression in healthy volunteers and patients with intracranial tumors or recurrent glioblastoma (NCT03539731) is ongoing. Shikonin, an inhibitor of PKM2, was reported to inhibit the proliferation of cancer cells and overcome chemotherapeutic drug-mediated resistance [102–104].

Dichloroacetate (DCA), an inhibitor of mitochondrial PDK1, switches metabolism from cytoplasmic glycolysis to mitochondrial glucose oxidation and has shown activity against several human cancers [105, 106]. DCA is already in clinical trials in head and neck carcinoma, glioblastoma and other recurrent brain cancers (NCT01386632, NCT01111097, NCT00540176). Moreover, a phase 1 trial of DCA indicated that DCA is safe, well-tolerated and feasible for use in adults with recurrent malignant brain tumors [107].

We previously stated that NOX4 deficiency results in reduced expression of CPT1A which then downregulates FAO, thereby inhibiting the activation of inflammasome. Moreover, a phase II clinical trial examined the effect of GKT137831, an inhibitor of NOX4, in patients with Primary Biliary Cholangitis (PBC), but until now, there have been no data available.

GLS, which controls glutamine metabolism, plays a vital role in up-regulating cell metabolism for tumor cell growth [108]. CB-839 is a potent, selective inhibitor of glutaminase which exhibits antiproliferative activity against various cancers in vitro and in vivo [109, 110], and it is currently being evaluated in several clinical trials.

Meanwhile, several therapeutic targets have been used to target upstream regulators of metabolic pathways, including HIF1α, mTOR and Akt.

HIF-1 is a transcription factor that regulates genes expression involved in glycolysis, angiogenesis, cell proliferation and survival [111]. PX-478 is an experimental HIF1α inhibitor that has shown antitumor activity against a variety of cancer cell lines under normoxia and hypoxia in vitro and in vivo [112, 113]. Moreover, PX-478 can enhance the radiosensitivity of prostate carcinoma cells [114]. Both phase 1 trial of oral PX-478 in patients with advanced solid tumors or lymphoma (NCT00522652) and a pilot study of EZN-2968(RO7070179), an antisense oligonucleotide inhibitor of HIF1α, in adults with advanced solid tumors (NCT01120288) have been completed. The latter was closed prematurely when the sponsor suspended the development of this agent, and the former results need to be posted further. Importantly, another clinical study showed that RO7070179 might benefit HCC patients and such activity needs to be further evaluated in a phase II study with more patients [115].

We previously introduced mTOR induces glycolysis, it is now evident that mTOR also stimulates cell growth, and dysregulated expression of mTOR underlies a variety of human diseases. In light of this evidence, it is clear that targeting mTOR could have important clinical benefits for cancer therapy [116]. Temsirolimus, a specific inhibitor of mTOR, has shown benefits in patients with different types of tumors in several clinical trials (NCT00065468 [117], NCT00117598 [118], and NCT01222715) [119]). Everolimus, another mTOR inhibitor, has also shown antitumor efficacy in various clinical trials as a single drug or in combination with other drugs (NCT01136733 [120], NCT01524783 [121], NCT01783444 [122]). The third most common mTOR inhibitor, ridaforolimus (deforolimus), has shown antitumor activity in hematological malignancies (NCT00086125) [123], metastatic endometrial cancer (NCT00122343 [124], NCT00739830 [124]) and sarcoma (NCT00538239 [125], NCT00093080 [126]). What’s noteworthy is that this agent showed toxicity and adverse side effects in several clinical trials, thus improving the tolerance of this class of drugs is important for future studies.

Akt can activate mTOR signaling, thereby regulating metabolism [127]. Previous studies showed that perifosine, an inhibitor of Akt, had limited efficacy used as monotherapy in a variety of tumors in clinical trials (NCT00590954 [128], Japic CTI-132287 [129], NCT01051557 [130]) [131–133], but exhibited promising results in combination with other drugs (NCT00401011, NCT01002248) [134–136].

Moreover, accumulating evidence has revealed that epigenetic modifications have potential for therapeutic exploitation. Analysis of the metabolome of trained monocytes showed that fumarate accumulation induces epigenetic reprogramming in monocytes by inhibiting KDM5 histone demethylases, increasing H3K4me3 and H3K27 acetylation and promoting trained immunity [137]. Considering these effects, demethylating agents may constitute potential new drugs for use in inflammatory diseases. In addition, therapeutic treatment targeting acetylation modification is also promising. An additional study showed that the histone acetyltransferase (HAT) p300, may link increased histone acetylation to transcriptional induction of some Akt-dependent M2 genes, as the p300 inhibitor C646 reduced the induction of Akt-dependent M2 genes [36]. However, one challenge is that the same pathway used by tumor cells and other cells in the TME may play opposing roles depending on the cellular context. A study conducted by Wenes et al. demonstrated that mTOR activation has antitumor effects in hypoxic TAMs but protumor effects in cancer cells. The antitumor effect of systemic mTOR inhibition occurs mainly through inhibition of this pathway in cancer cells but is partly countered by the effect on TAMs. In this setting, pharmacological inhibition of mTOR in combination with TAM-depleting strategies exhibited enhanced effects. Moreover, the development of new nanomedicines to target and impact TAMs might be another promising option to successfully treat tumors [138].

## Conclusion and perspectives

The investigation of immunometabolism has led to several key discoveries regarding the interplay between metabolism and cell function. As mentioned above, metabolic disparities exist among macrophages. Studies conducted by Artyomov et al. showed that mitochondrial OXPHOS is increased in LPS-activated pMACs compared with BMDMs under the same stimulation conditions and that LPS-activated pMACs exhibit delayed induction of iNOS and increased arginase expression, which suppresses NO generation and lessens ETC inhibition [5]. Interestingly, different stimuli can lead to different metabolic characteristics. Studies have indicated that a shift from OXPHOS to glycolysis is observed in monocytes costimulated with TLR4 and LPS but not in monocytes stimulated by other TLRs, such as TLR2. Activation with Pam3CysSK4 (P3C) or other bacterial lysates and transcriptome and metabolomic analyses have shown that compared with monocytes stimulated with LPS, monocytes stimulated with P3C exhibit significant differences in the TCA cycle, OXPHOS and lipid metabolism pathways [6]. In addition, proinflammatory stimulation of macrophages in mouse models has been shown to lead to iNOS expression and NO release, while human macrophages produce only low levels of NO in vitro. Moreover, iNOS activity in monocytes is not altered by treatment with LPS with or without IFN-γ [7, 8, 139]. Whether these differences exist remains to be investigated.

In addition, the aforementioned observations indicate that inhibition of glycolysis or FAO by 2-DG or etomoxir has off-target effects; therefore, the observed results using these agents to inhibit glycolysis or FAO need to be verified by genetic approaches. Strikingly, determining whether an observed metabolic shift has a correlative role or a causal role in macrophage functions is often difficult. In this sense, inhibition of related metabolic pathways is necessary.

Moreover, succinate, a metabolite of the TCA cycle, can increase the succinylation of several proteins, such as malate dehydrogenase, GAPDH, glutamate carrier 1, and LDH; however, the specific function of this modification requires further investigation [53]. In particular, the novel functions of the metabolites fumarate, citrate and itaconate in macrophages need to be explored under different conditions.

Importantly, although the current literature regarding TAM metabolism is relatively limited, the available studies suggest that the levels of macrophage metabolism are often abnormal in disease states. Therapies targeting macrophage metabolism have intriguing potential, and further research is urgently needed.

Ultimately, several questions on the development of targeting metabolic pathways for cancer treatment remain unanswered. First, as normal cells have the same metabolic requirements as cancer cells, how to find a therapeutic window during treatment. Second, both tumor cells and TAMs exhibit metabolic heterogeneity, how to find efficiently targets that are efficacious and specific for tumors with minimal toxicity. Finally, can we stratify patients based on genetics when developing metabolism-targeted drugs.

## Data Availability

The material supporting the conclusion of this review has been included within the article.
